# Spinal accessory nerve neuropathy following neck dissection

**DOI:** 10.1590/S1808-86942011000200017

**Published:** 2015-10-19

**Authors:** Luciana Pereira de Lima, Ali Amar, Carlos Neutzling Lehn

**Affiliations:** 1 Physical therapist, Intern - Head and Neck Surgery Department - Hospital Heliópolis; 2 PhD. Head and Neck Surgeon - Hospital Heliópolis; 3 PhD. Head and Neck Surgeon - Hospital do Servidor Público Estadual. Hospital Heliopolis

**Keywords:** cranial nerves/abnormalities, electromyography, neck dissection

## Abstract

The most common complication of neck dissection is shoulder dysfunction due to manipulation of spinal accessory nerve, resulting in trapezius muscle atrophy mainly in procedures involving the posterior neck triangle.

**Aim:**

This study used electromyography to evaluate the injury to the spinal accessory nerve following neck dissection.

**Materials and methods:**

Prospective case series of 51 patients submitted to 60 neck dissections followed by physical therapy evaluation of shoulder dysfunction. Nerve integrity was evaluated before and after the surgery by means of surface EMG registering the electric activity of the trapezius muscle during voluntary contraction. The patients were grouped according to the type of neck dissection, presence of shoulder pain, impairment during abduction movement and hypotrophy/ atrophy of the trapezius muscle.

**Results:**

Action potential had median values of 54.3 microV before surgery and 11.6 microV after it (*p*<0.001). There was a mean decrease of 70% comparing to preoperative values. The median was 12.5 microV after dissection including level IIb, and 8.9 microV after dissection including levels IIb and V (*p*<0.002).

**Conclusion:**

Surface EMG is a sensitive and painless method for spinal accessory nerve dysfunction evaluation. The results suggest the usefulness of the trapezius muscle electromyography to confirm diagnosis and early physical therapy intervention in neuropathies of the spinal accessory nerve.

## INTRODUCTION

The main prognostic factor associated with head and neck cancer is the presence of lymph node metastasis in the neck, and neck dissection (ND) is the gold standard treatment for such metastases. ^1^ However, this procedure may cause severe morbidity. One of the most common complications stemming from ND is shoulder dysfunction caused by manipulation of the spinal accessory nerve (XI cranial nerve) - which causes atrophy of the trapezium muscle.

Spinal nerve injuries may be missed during clinical exam and are only discovered by an electromyography test. Electromyography (EMG) has shown that the trapezium muscle is the main muscle responsible for shoulder elevation and, by means of its upper bundle; it participates in the arm elevation movement^2^. Nonetheless, this movement also involves the participation of the deltoid, supra-spinal and infra-spinal muscles. For these reasons, arm elevation paresis secondary to spinal nerve mononeuropathy may be missed by clinicians, because the movement may be compensated by the action of the other muscles responsible for arm elevation^3^.

Studies which assess superficial muscles have used surface electromyography (EMG) to collect data, studying its clinical application, since it is a non-invasive method, safe and easy, which does not cause discomfort and enables one to quantify muscle electrical activity^4^.

Very few studies have used EMG to do an early assessment of shoulder dysfunction after ND. Thus, the aim of the present study is to present the clinical and electrophysiological data from 51 patients submitted to neck dissection surgery to treat head and neck neoplasias.

## MATERIALS AND METHODS

This is prospective series involving 51 patients submitted to 60 neck dissections to treat head and neck malignant neoplasia. We took off this study the patients who had been previously irradiated, with peripheral nerve damage prior to surgery, pain or previous trauma in the ipsilateral shoulder or post-operative complications which prevented evaluation 30 days after the procedure.

We used a specific protocol, with pre-op and postop physical evaluation, including patient age, gender, primary site, surgery performed and types of neck dissection with level and sublevel descriptions. All the patients selected were told about the study and singed a free and informed consent form.

They were submitted to clinical evaluation and EMG study of their sensitive and motor neuroconduction, by a qualified professional in the pre-op and postoperative periods (21 to 30 days after the procedure) before the patient was referred to adjuvant radiotherapy.

EMG exams were carried out with the patient seating down, two electrodes were fixed to the skin, in the thickness of the upper muscle belly of the trapezium, placed in the middle point, following recommendations from SENIAM - *European recommendations for surface electromyography*, available online at http://www.seniam.org.

We used Meditrace® 200 surface leads of Ag/AgCl with disposable gel-loaded conduction adhesive. The action potentials (electrical activity) of the trapezium muscle motor units were recorded during maximum isometric muscle contraction (MIMC) in three Five-second series, with a five second interval between each series, according to the technique described by De Luca^5^. Of the electromyographic signal from the device used was the Miotol 400, from Miotec®, with 4 channels, using the Miograph, version 2006 software. In the motor neuroconduction study carried out by EMG, for signal acquisition, we used as a reference for normalization, the collection of values from the median in *Root Mean Square* (RMS) of the MIMC electromyographic signal^4^. The data is presented in microvolts (mv), after using a band pass filter of 20 to 500 Hz, provided by the conversion software that comes with the equipment.

In the clinical exam we considered the presence/absence of pain, using the analogue-visual scale (AVS). In the trapezium we observed the presence/absence of atrophy/hypotrophy or paralysis (a specific test for the trapezium muscle); the arm active range of motion was observed for higher/lower than 90 degrees opening (arm active abduction test), confirmed with a universal goniometer device.

Results were evaluated according to the extension of accessory nerve manipulation, broken down into two groups: group 1 (IIb dissection level) with minimum nerve manipulation; and group 2 (IIb and V dissection levels), major nerve manipulation.

The statistical analysis employed non-parametric methods, with the Mann-Whitney and Wilcoxon tests for the quantitative variables and the Yates-corrected chisquared test for the qualitative variables, and *p* values below 0.05 were considered significant.

The present study was approved by the Ethics in Research Committee of our institution.

## RESULTS

In the series studied, 50 (83%) patients were males and e 10 (16%) were females, with ages varying between 26 and 73 years (mean of 53 years).

In the postoperative evaluation, pain was reported by all patients and hypotrophy/atrophy or paralysis was also found in all cases.

**Figure 1:**
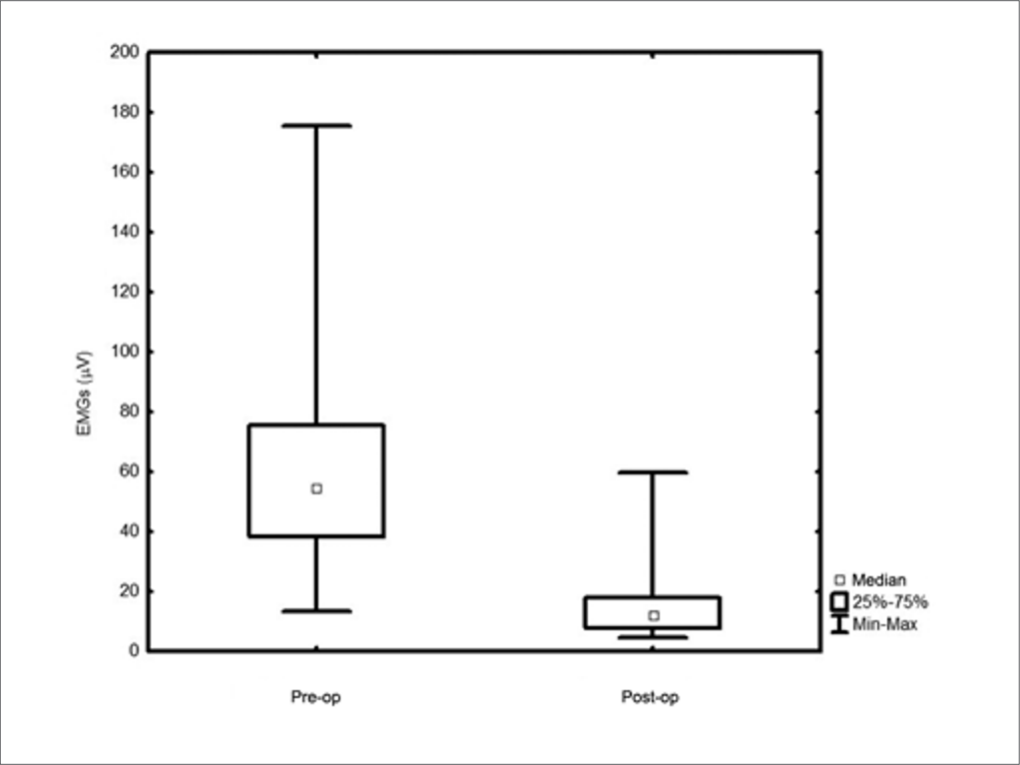
Pre-op and postoperative electromyography - *p*=0.001

**Figure 2:**
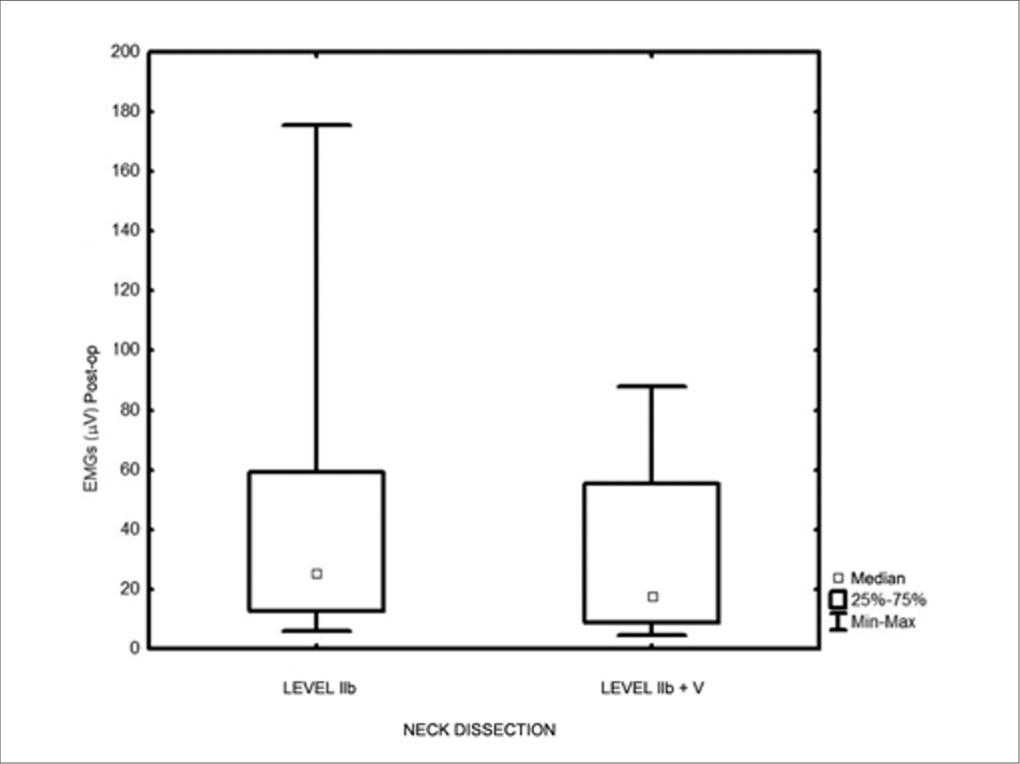
Electromyography in relation to the dissection extension - *p*=0.002.

**Figure 3:**
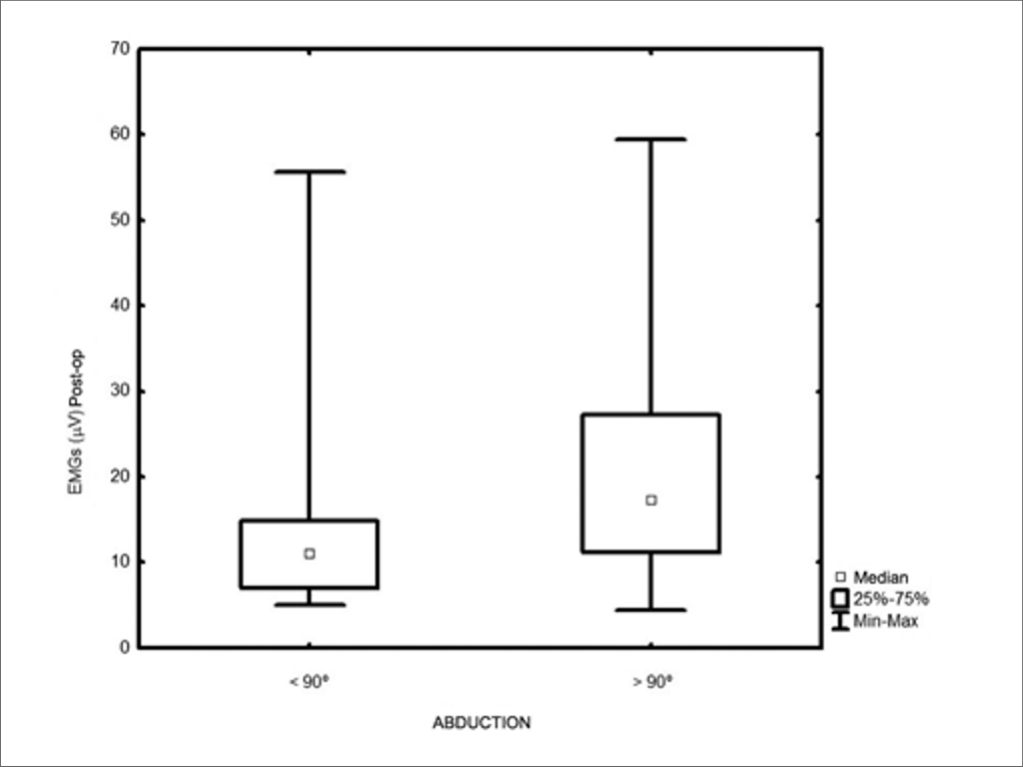
Electromyography in relation to the arm movement. - *p*=0.01.

The action potential showed a median value of 54.3 μV (13.3 to 175.3 μV) in the preoperative; and 11.6 μV (4.3 to 59.4 μV) in the postoperative (*p*<0.001). As to the ND extension, we found median values of 12.5 mV after level IIb dissection and 8.9 mV with dissections levels IIb+V. (*p*=0.002). There was a major variation in the action potentials recorded both during pre-op and in the post-op (Fig. 1). The post-op values had a mean reduction of 70% (20% to 94%) when compared to preoperative values. As to the dissection extension, 90% (19/21) of the cases submitted to a level V dissection had upper limb abduction lower than 90 degrees, while only 56% (22/39) of the patients with level IIb manipulation only had shoulder movement restriction of this magnitude (*p*=0.01). We noticed lower action potentials in the patients with levels IIb and V neck dissections, as well as in those with abduction lower than 90 degrees (Figs. 2 and 3), although there is much value overlapping.

## DISCUSSION

The consensus adopted by the *American Academy of Otolaryngology Head and Neck Surgery* and recommended by the *American Head and Neck Society* on the classification and terminology used for neck dissections encourages the use of a new nomenclature, described as: radical neck dissection (RND), modified radical neck dissection (MRND) (with specification of the removed structures), selective neck dissection (SND) (with the specification of the levels and sublevels which were removed), and broad neck dissection (BND)^6^. When properly planned, more conservative procedures such as the MRND and the SND respect the principles of oncologic radicalism, besides having the additional advantage of minimizing the functional deficiencies resulting from RND and BND. The most relevant functional aspect is, without doubts, the involvement of shoulder function arising from damage to the XI cranial nerve with consequent trapezium muscle denervation^7^. The anatomical characteristics of the spinal accessory nerve explain why surgical procedures in the neck, especially those done in the posterior triangle, frequently cause paralysis to this nerve^3^.

The XI cranial nerve is formed by a cranial root and a spinal root. The common trunk crosses the jugular foramen, together with the glossopharyngeal and vagus nerves, dividing itself into an internal and one external branch. The internal branch joins the vagus and goes along with it. The external branch has the spinal root fibers, it has its own route and moves obliquely downwards and to the back, innervating the trapezium and the sternocleidomastoid muscles. The accessory nerve may be joined by the deep neck plexus of the sternocleidomastoid muscle; however, its motor contributions remain uncertain^8^, ^9^.

The shoulder syndrome, resulting from RND, was first described by Erwing and Martin in 1952 and the term was concocted by Nahum et al. in 1961. Its clinical manifestations include constant pain, shoulder tilt and drop, difficulties in shoulder retraction, limitations in the anterior flexion movements and active shoulder abduction, winged scapula and abnormal electromyographic findings^10^, ^11^.

Cappiello et al. Observed that the MRND increase shoulder morbidity when compared to SND^12^. On the other hand, Koybasioglu et al. reported that the accessory nerve function is better in MRND when compared to the lateral ND, because of the traction applied to the nerve during sternocleidomastoid muscle retraction, in order to expose the surgical field^13^. Another study led by Tsuji et al. also confirms the complete or incomplete denervation of the trapezium muscle caused by the axonal injury to the XI cranial nerve, even if it is preserved, because of the traction caused to the accessory nerve during ND^14^. In our study, all the patients had a decrease in post-op electrical activity, with a significant difference in the group in which sublevel IIb was added to level V.

Recent studies have confirmed in their electromyographic findings the deterioration which happens in immediate post-op and the gradual improvement which happens in the subsequent months after surgery; however, without recovery of the original function of the accessory nerve. Electrophysiological evaluations have shown that, despite the nerve's anatomical integrity, the risk is even greater whenever the neck's posterior triangle is involved (level V)^1^’^15^, ^16^, ^17^.

The muscles’ denervation potentials become characteristic after two to three weeks of the lesion, first on the proximal muscles, later on the distal ones. Therefore, the test can result false negative if done early on. In our study we found a reduction in the electrical activity of the descending fibers of the trapezium muscle after surgery, with great variability among the individuals; this happens because of variables such as BMI, age and prior physical activities, justifying the use of EMGs in the clinical assessment of the individual.

Inoue et al. reported that the inclusion of level V in the procedure worsens quality of life when compared to ND with the preservation of the accessory nerve. The cutting of the sternocleidomastoid muscle and/or that of the spinal accessory nerve had a significant impact on daily activities, work and leisure activities^18^. Dijkstra et al. reported that the shoulder pain was clinically present in 70% of the patients after the ND surgery^19^. Cheng et al. reported that 100% had shoulder pain and that 80% had shoulder drop after the RND surgery. These findings are in agreement with those from our study^15^.

We have noticed that SND causes better shoulder function when compared to other types of dissections; this difference has been explained by less level V manipulation during the surgical procedure, resulting in less damage to the accessory nerve and the neck plexus^1^, ^7^, ^12^, ^13^, ^14^, ^17^, ^18^.

The shoulder syndrome is an important sequela of ND. Although not being paramount for the diagnosis and treatment of shoulder dysfunction, EMG is a quantitative method which enables us to better assess the effects of accessory nerve manipulation and, especially, to assess different rehabilitation strategies. Nonetheless, further studies are still needed in order to better guide its clinical application.

## CONCLUSION

In our study, EMG proved to be a sensitive and painless method used to help in the early diagnosis of XI cranial nerve dysfunction. In the post-op, all the patients had an impairment of the upper bundle of the trapezium muscle, with pain and abduction movement limitation of that arm. Our findings suggest the benefit of using EMG in the trapezium to confirm the diagnosis and to guide early physical therapy intervention in probable neuropathies of this nerve.
